# Epidemiology of *Coxiella burnetii* Infection in Africa: A OneHealth Systematic Review

**DOI:** 10.1371/journal.pntd.0002787

**Published:** 2014-04-10

**Authors:** Sky Vanderburg, Matthew P. Rubach, Jo E. B. Halliday, Sarah Cleaveland, Elizabeth A. Reddy, John A. Crump

**Affiliations:** 1 Duke Global Health Institute, Duke University, Durham, North Carolina, United States of America; 2 Division of Infectious Diseases and International Health, Department of Medicine, Duke University Medical Center, Durham, North Carolina, United States of America; 3 Institute of Biodiversity, Animal Health and Comparative Medicine, University of Glasgow, Glasgow, Scotland, United Kingdom; 4 Kilimanjaro Christian Medical Centre, Moshi, Tanzania; 5 Kilimanjaro Christian Medical University College, Moshi, Tanzania; 6 Centre for International Health, Dunedin School of Medicine, University of Otago, Dunedin, New Zealand; Centers for Disease Control and Prevention, Kenya

## Abstract

**Background:**

Q fever is a common cause of febrile illness and community-acquired pneumonia in resource-limited settings. *Coxiella burnetii*, the causative pathogen, is transmitted among varied host species, but the epidemiology of the organism in Africa is poorly understood. We conducted a systematic review of *C. burnetii* epidemiology in Africa from a “One Health” perspective to synthesize the published data and identify knowledge gaps.

**Methods/Principal Findings:**

We searched nine databases to identify articles relevant to four key aspects of *C. burnetii* epidemiology in human and animal populations in Africa: infection prevalence; disease incidence; transmission risk factors; and infection control efforts. We identified 929 unique articles, 100 of which remained after full-text review. Of these, 41 articles describing 51 studies qualified for data extraction. Animal seroprevalence studies revealed infection by *C. burnetii* (≤13%) among cattle except for studies in Western and Middle Africa (18–55%). Small ruminant seroprevalence ranged from 11–33%. Human seroprevalence was <8% with the exception of studies among children and in Egypt (10–32%). Close contact with camels and rural residence were associated with increased seropositivity among humans. *C. burnetii* infection has been associated with livestock abortion. In human cohort studies, Q fever accounted for 2–9% of febrile illness hospitalizations and 1–3% of infective endocarditis cases. We found no studies of disease incidence estimates or disease control efforts.

**Conclusions/Significance:**

*C. burnetii* infection is detected in humans and in a wide range of animal species across Africa, but seroprevalence varies widely by species and location. Risk factors underlying this variability are poorly understood as is the role of *C. burnetii* in livestock abortion. Q fever consistently accounts for a notable proportion of undifferentiated human febrile illness and infective endocarditis in cohort studies, but incidence estimates are lacking. *C. burnetii* presents a real yet underappreciated threat to human and animal health throughout Africa.

## Author Summary

*Coxiella burnetii* is a bacterium that can cause acute and chronic fever illness and pneumonia in humans. It is also a known cause of abortion in livestock species, and is principally transmitted to humans through contact with infected animal birth products. With growing awareness of the over-diagnosis and misclassification of malaria as the cause of fever illnesses in the tropics, including Africa, there is increased interest in the role of non-malarial causes of fever, such as *C. burnetii*. We performed a systematic review of the published literature on the epidemiology of *C. burnetii* in Africa to consolidate knowledge and identify knowledge gaps regarding the extent of this infection in humans and animals and the risk factors for infection transmission. Few studies on prevalence of infection in humans and animals used random sampling strategies, and among these only two studied linked human and animal populations. *C. burnetii* appears to be a common cause of severe fever illness in humans, but population-level incidence estimates are lacking. The differential risks for *C. burnetii* infection and potential control strategies within the various animal husbandry systems in Africa remain largely unexplored. We conclude that *C. burnetii* is an underappreciated threat to human and animal health throughout Africa.

## Introduction

*Coxiella burnetii*, a zoonotic bacterial pathogen found worldwide except in New Zealand, is transmitted to humans through direct contact with milk, urine, feces, or semen from infected animals as well as inhalation of aerosolized particles from animal placentas, parturient fluids, aborted fetuses, and environmental dust [1]. While infection by *C. burnetii* in humans can be asymptomatic, symptomatic infection, known as Q fever, can present as an acute undifferentiated febrile illness with the possibility of focal manifestations, such as hepatitis and pneumonia. Acute disease can progress to chronic forms, such as endocarditis, in 0.5–2.0% of cases [2], [3], typically in individuals predisposed by valvular heart disease or immunodeficiency [4]. Q fever is also one of the infectious diseases that has been linked to chronic fatigue syndrome [5]. Infection by *C. burnetii* has been demonstrated in many animal species, but the principle reservoirs are thought to be sheep, goats, and cattle. In these livestock species infection is often asymptomatic but can cause abortion and reduce reproductive efficiency [1], [6].

Q fever has recently gained renewed attention after the largest-ever recorded outbreak which involved over 3,500 human cases in the Netherlands in 2007–2009 [7]. Recent studies in resource-limited settings have demonstrated *C. burnetii* as a common cause of febrile illness and community-acquired pneumonia [8]–[10]. Fever etiology research among hospitalized patients in northern Tanzania found Q fever was a more common cause of severe febrile illness than malaria [11], [12]. As control efforts have led to consistent decreases in malaria incidence throughout sub-Saharan Africa [13]–[17] the diagnosis, treatment, and control of non-malaria febrile illnesses, such as Q fever, are emerging as new public health priorities [12]. In addition to being sources for disease transmission to humans, *C. burnetii* infection in animals can decrease livestock productivity which can have socioeconomic and indirect health effects on humans, especially among livestock-keeping populations in resource-limited settings [18].

In light of recent findings establishing Q fever as an important cause of severe febrile illness in northern Tanzania [11], [12] and growing awareness of the potential economic impact of infection in animals, we systematically reviewed the existing literature on the epidemiology of *C. burnetii* infection among humans and animals in Africa. This survey aimed to consolidate knowledge and identify future research priorities for the following topics: the prevalence of *C. burnetii* infection in human and animal populations, including surveys of sera or shedding in body fluids; the incidence of disease due to *C. burnetii* in human and animal populations; risk factors for seropositivity or disease; and infection control efforts undertaken in Africa.

## Methods

### Search

**Figure 1:**
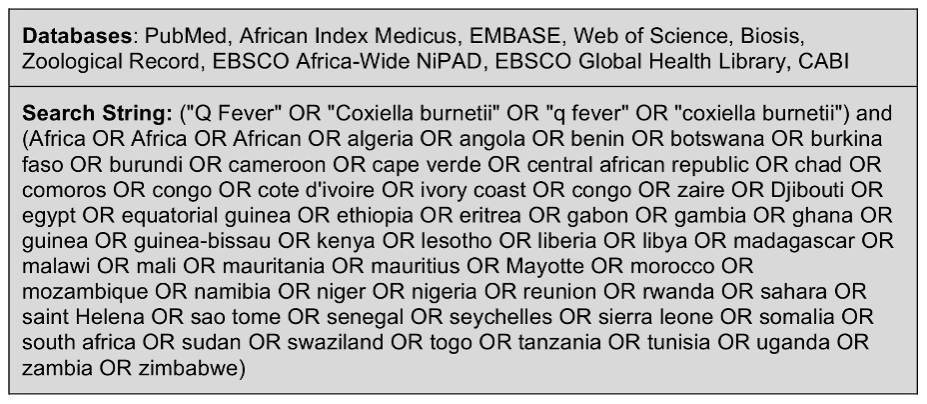
Databases and search terminology employed for systematic review of *Coxiella burnetii* epidemiology in Africa, all years.

Nine databases were searched with the search string described in **Figure 1** including all countries in the 5 United Nations (UN) sub-regions of Africa [19]. These search terms were adapted to the particular language of each database, and for those databases that did not allow the combination of Boolean operators, (q fever) OR (*Coxiella burnetii*) was used. Two of the databases, CABI and EBSCO Global Health, were searched with the intention to include grey literature.

### Selection and data extraction

**Figure 2:**
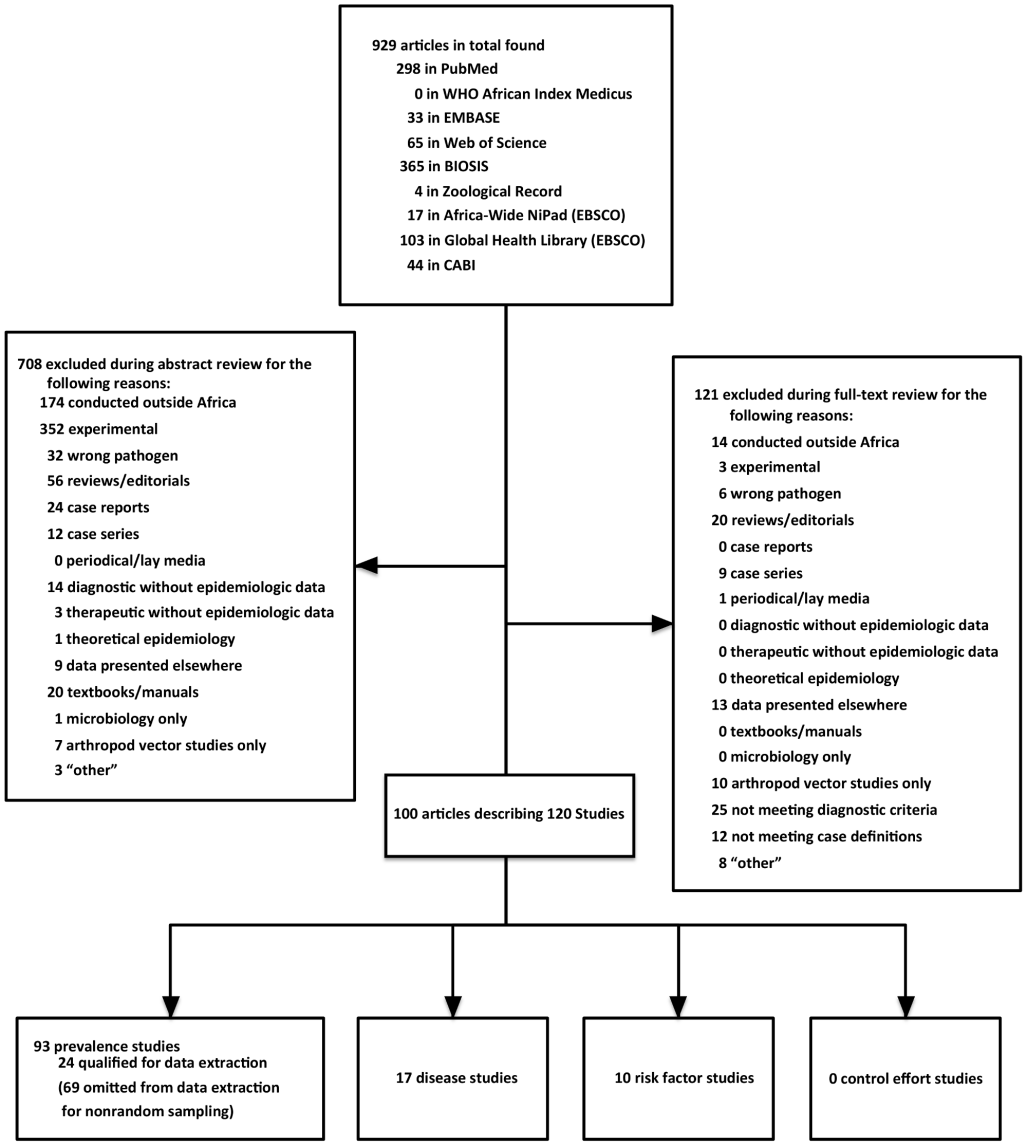
Selection of eligible articles concerning *Coxiella burnetii* epidemiology in Africa.

Citations for all years and in all languages were compiled and de-duplicated using EndNote (Thomson Reuters, New York, USA). Abstracts were independently reviewed by two investigators (SV and MPR) using combined language competency in English, French, Spanish, and Portuguese or Google Translate (Google, Mountain View, CA, USA) and included for full-text review upon meeting predetermined criteria (**Figure 2**). Excluded abstracts described studies conducted outside Africa, basic science or immunology experiments, incorrect pathogens, reviews/editorials, case reports or case series, Q fever among returning travelers, periodical lay media content, diagnostic or therapeutic studies without epidemiologic data, theoretical epidemiology, duplicate data published elsewhere, textbooks/manuals, microbiologic studies without epidemiologic data, or arthropod sampling. The same criteria were applied during full-text review and to all grey literature containing sufficient information for adjudication. Cases of disagreement between the two investigators were resolved through independent review by a third investigator (JEBH).

### Prevalence of *Coxiella burnetii* infection

Prevalence studies that presented evidence of current or prior infection with *C. burnetii* in humans and animals were included. We considered the following serologic tests and minimum antibody titer cut-offs for *C. burnetii* phase I and/or phase II antigen as acceptable evidence of infection in humans and animals based on expert consensus: complement fixation (CF) >1∶10 for animals [20] and ≥1∶4 for humans, microscopic agglutination test (MAT) ≥1∶4 for humans and animals, indirect fluorescent antibody (IFA) ≥1∶25 for animals and ≥1∶40 for humans, and ELISA validated against one of the above methods [20]–[23]. Capillary agglutination tests (CAT) were also accepted based on demonstrated correspondence to CF titers [23], [24]. The terms seropositive or seropositivity are used throughout to describe serologic reactions that met these titer cut-offs. For studies of pathogen shedding, confirmation of *C. burnetii* by nucleic acid detection, culture, or rodent inoculation was accepted. Studies of the prevalence of *C. burnetii* infection in humans and animals were classified by extent of study population characterization and sampling strategies, which were categorized as random (e.g., proportional, simple cluster, or simple random) or non-random. Prevalence studies that met these diagnostic criteria and used randomized sampling strategies qualified for data extraction. Prevalence studies with appropriate diagnostic criteria that used non-random sampling were included but did not undergo further data extraction.

### Disease attributed to *Coxiella burnetii*

Studies reporting disease in animals due to *C. burnetii* were included if the reported cases met the World Organization for Animal Health (OIE) case definition for Q fever: abortion and/or stillbirth plus confirmed presence of the bacterial agent, accomplished by 1) isolation in culture; or 2) polymerase chain reaction (PCR), *in situ* hybridization, or immunohistochemistry of birth products or of associated vaginal discharge [20]. Evidence of *C. burnetii* on placental smears with stains deemed appropriate by OIE (Stamp, Ziehl-Neelsen, Gimenez, Giemsa or modified Koster) were considered presumptive for disease and included [20]. We also included seroprevalence studies of animals with a history of abortion, as these data, although not of confirmed cases, could yield information about potential associations between *C. burnetii* exposure and prevalence of animal abortion.

For studies of disease in humans, acute Q fever was defined according to the US Centers for Disease Control and Prevention (CDC) case definition: a compatible fever syndrome plus four-fold rise in antibody titers to Phase II antigen or detection in clinical specimens by PCR, immunohistochemistry (IHC), or culture [25]. Phase II antigen IFA antibody titer levels for IgG≥1∶200 and IgM≥1∶50 were included as cases based on the high positive predictive value of such results [26]. Studies reporting chronic disease in humans due to Q fever were included if the reported cases met the CDC case definition for confirmed chronic Q fever: culture-negative endocarditis or infected vascular aneurysm, chronic hepatitis, osteomyelitis, osteoarthritis, or pneumonitis with no other etiology plus IFA IgG antibody to *C. burnetii* phase I antigen ≥1∶800 or detection in clinical specimens by PCR, IHC, or cell-culture [25].

### Risk factors for *Coxiella burnetii* infection or disease

Risk factor studies were evaluated using the same criteria for sampling design and case definitions that were applied to surveys of prevalence or disease, respectively. Risk factor analyses in prevalence studies were excluded if the prevalence study used a non-random sampling strategy.

### *Coxiella burnetii* control efforts

Studies describing control efforts must have presented original data demonstrating the outcome of an intervention to decrease infection or disease incidence in human and/or animal populations.

### Data extraction and analysis

For all qualifying studies, extracted data included study country, city or region, species, population census data when given, sample size, year of study, and diagnostic test as well as the number of seropositive or disease cases, risk factors, or control effort data where applicable. In the case of incomplete data or unclear methods, authors were contacted for further clarification when possible. Descriptive analyses of the extracted data were conducted. No quantitative meta-analysis was undertaken.

## Results

**Figure 3:**
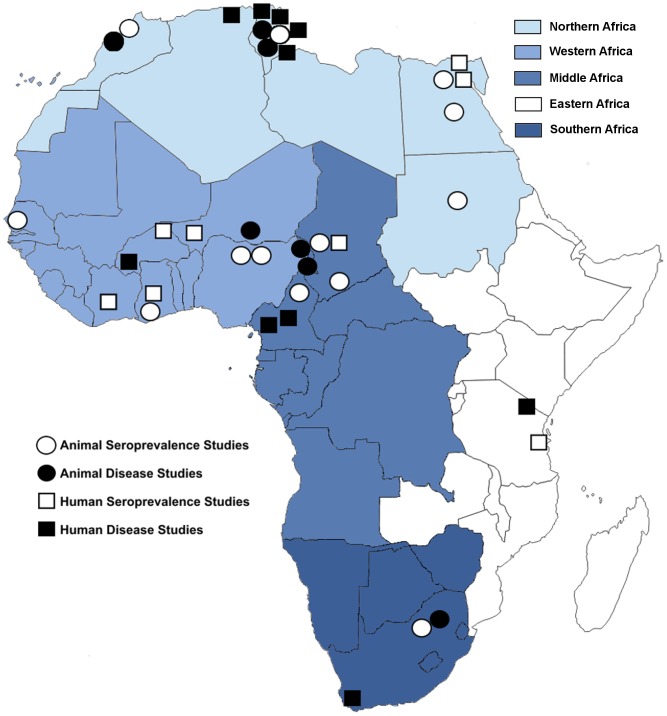
Locations of *Coxiella burnetii* infection prevalence and disease studies in African humans and animals which qualified for data extraction.

A total of 1,662 citations were identified by the search conducted on December 3, 2012. After duplicates were removed, 929 articles remained (**Figure 2**). Four of the six authors we contacted for further clarification responded to our inquiries. Ultimately, 100 articles describing 120 studies remained after full-text review. Forty-one articles describing 51 studies qualified for data extraction. The other 59 articles described 69 prevalence studies that did not qualify for data extraction due to non-random sampling methods (**Table S1**). Studies qualifying for data extraction were grouped into the following categories: 8 human and 13 animal seroprevalence; 3 animal milk shedding; 10 human disease; 7 animal abortion; and 7 human and 3 animal risk factor studies. These 51 studies spanned the years 1965–2012 and were conducted in 15 countries, mostly concentrated in the UN sub-regions of Northern, Western, and Middle Africa (**Table 1** & **Figure 3**).

**Table 1:** Studies of *Coxiella burnetii* infection prevalence and disease prevalence in humans and animals in Africa that qualified for data extraction.

Sub-Region	Study Type	Country	Species (n)	Study Year(s)	Sample	Number Positive (%)	Ref
**Northern**	Prevalence	Egypt	Dogs (150)	1998–1999	serum	34 (23%)	[37]
		Egypt	Buffalo (45)	2012*	serum	0 (0%)	[27]
			Cattle (54)			7 (13%)	
			Goats (30)			7 (23%)	
			Sheep (55)			18 (33%)	
			Humans (92)			15 (16%)	
		Egypt	Cattle (100)	2009*	milk	22 (22%)	[38]
		Egypt	Humans (883)	1991	serum	285 (32%)	[39]
		Sudan	Goats (460)	2010–2011	serum	109 (24%)	[36]
	Disease	Morocco	Sheep prior abortion (115) and controls (156)	1986–1987	serum	38 (33%) and 23 (15%)	[48]
		Tunisia	Goats & Sheep prior abortion (118) and Sheep controls (517)	1997	serum	22 (12%) and 35 (7%)	[49]
		Tunisia	Goats & Sheep abortion (72)	2009*	birth products/vaginal fluids	14 (19%)†	[45]
		Tunisia	Human febrile illness (300)	1993–1994	serum	5 (2%)	[54]
		Tunisia	Human febrile illness (47)	2004	serum	4 (9%)	[55]
		Tunisia	Human endocarditis (98)	1991–2000	blood	1 (1%)	[51]
		Tunisia	Human endocarditis (33)	2008	serum	1 (3%)	[52]
		Algeria	Human endocarditis (61)	2000–2003	serum	2 (3%)	[53]
**Western**	Prevalence	Nigeria	Cattle (306) Cattle (84)	1983–1984	serum milk	169 (55%) 44 (52%)	[30]
		Nigeria	Cattle (88) Cattle (169)	1983–1984	serum milk	48 (55%) 41 (24%)	[31]
		Senegal	Cattle (196)	2007–2008	serum	7 (4%)	[29]
		Ghana	Cattle (166)	2012*	serum	30 (18%)	[32]
		Cote d'Ivoire	Humans (949)	1965	serum	44 (5%)	[42]
		Burkina Faso	Humans (1309)	1975*	serum	100 (8%)	[43]
		Niger	Humans, children (177)	1994	serum	17 (10%)	[40]
		Ghana	Humans, children (219)	2008*	serum	37 (17%)	[41]
	Disease	Niger	Goats prior abortion (75) and controls (47)	1971–1972	serum	24 (32%) and 12 (29%)	[47]
		Burkina Faso	Human febrile illness (183)	1995	serum	9 (5%)	[56]
**Middle**	Prevalence	Chad	Cattle (193)	1985*	serum	13 (7%)	[34]
		Chad	Camels (142)	1999–2000	serum	114 (80%)	[28]
			Cattle (195)		serum	8 (4%)	
			Goats (134)		serum	18 (13%)	
			Sheep (142)		serum	16 (11%)	
			Humans (368)		serum	4 (1%)	
		Cameroon	Cattle (1377)	2000	serum	431 (32%)	[33]
	Disease	Cameroon	Cattle prior abortion (116)	1968*	serum	0 (0%)	[50]
		Cameroon	Cattle prior abortion (490) and controls (193)	1985*	serum	14 (3%) and 13 (7%)	[34]
		Cameroon	Human pneumonia (110)	1991–1992	serum	6 (6%)	[57]
		Cameroon	Human pneumonia (65)	1991–1993	serum	6 (9%)	[58]
**Southern**	Prevalence	South Africa	Cattle (8900)	1985–1986	serum	692 (8%)	[35]
	Disease	South Africa	Cattle (6 farms) and sheep (6 farms) abortions	1972–1976	fetal tissue	12 (100%) farms+	[46]
		South Africa	Human pneumonia (92)	1987–1988	serum	0 (0%)	[59]
**Eastern**	Prevalence	Tanzania	Humans, pregnant females (150)	1993	serum	7 (5%)	[44]
	Disease	Tanzania	Human febrile illness (483)	2007–2008	serum	24 (5%)	[11]

*Year(s) of sample collection for study unavailable; year of publication included instead.

+ *C. burnetii* detection by impression smear microscopy. Positive results were not provided at an individual animal level, only at the level of investigated farms.

† *C. burnetii* detection by polymerase chain reaction.

### Prevalence of *Coxiella burnetii* infection

Two surveys employed a systematic sampling strategy to assess seroprevalence among linked human and animal populations. The first, from three governorates surrounding Cairo, Egypt, reported *C. burnetii* seropositivity in 13% of cattle, 23% of goats, 33% of sheep, 0% of buffalo, and 16% of humans in close contact with these animals [27]. The second survey, undertaken in Chad, found 80% of camels, 4% of cattle, 13% of goats, 11% of sheep, and 1% of humans in close contact were seropositive [28]. All other seroprevalence studies sampled only humans or only animal species (**Table 1**).

Surveys of cattle demonstrated seroprevalence ranging from 4% in Dakar, Senegal [29], to 55% around the city of Zaria, Nigeria [30], [31]. Other studies reporting cattle seroprevalence within this range were conducted in coastal Ghana (18%) [32], Cameroon's Adamawa Region (32%) [33], southern Chad (7%) [34], and South Africa's Transvaal Province (8%) [35]. Goat seropositivity ranged from 13% in Chad [28] to 23% in Egypt [27] and 24% in 8 Sudanese states [36]. Surveys of sheep revealed seroprevalences that ranged from 11% in Chad [28] to 33% in Egypt [27]. In Upper Egypt, 23% of dog sera samples indicated prior *C. burnetii* infection [37].

In studies of pathogen shedding in bovine milk, *C. burnetii* nucleic acid was detected in 22% of raw milk samples in Upper Egypt [38]. In Zaria, Nigeria, *C. burnetii* shedding among individual cows was reported in 63% of milk samples from extensively managed cattle and 43% of samples from semi-intensively managed cattle [30], whereas the prevalence was 26% and 22%, respectively, at the same location one year later [31]. No studies of shedding in fluids other than milk in asymptomatic humans or animals were found by our search, and no human milk shedding studies qualified for data extraction.

Seroprevalence in humans ranged from 1% in Chad [28] to 32% in a Nile Delta village in Egypt [39]. In Niamey, Niger, 10% of children ages 1 month-5 years were seropositive [40], and in Ghana's rural Ashanti Region, 17% of two-year-olds were seropositive [41]. Other surveys reported human seroprevalence at 5% in rural western Côte d'Ivoire [42], 8% among nomads sampled in rural northern Burkina Faso [43], and 5% of pregnant women attending an antenatal clinic in Dar es Salaam, Tanzania [44].

### Disease attributed to *Coxiella burnetii*

Of disease studies in humans or animals, none estimated disease incidence, and two studies [45], [46] of livestock abortions met OIE definitions for either presumptive or confirmed cases. The remaining 5 animal abortion studies were serological investigations in individuals with history of abortions [34], [47]–[50].

Of two surveys of cattle with abortions in northern Cameroon, one did not detect serological evidence of *C. burnetii* infection in any cattle [50], while 3% in the other study were seropositive, compared to 7% among a non-random selection of cattle without abortions [34]. In South Africa, *C. burnetii* was found by smear microscopy in aborted calf fetuses at all of six cattle farms sampled [46].

In the Maradi Region of Niger, 32% of goats with previous abortions were seropositive, compared to 29% of non-randomly selected goats without a history of abortion [47]. Sheep with a history of abortion in Rabat, Morocco, were more likely than those with normal births to be seropositive for *C. burnetii*, 33% versus 15% (p<0.01) [48]. In a survey conducted in five Tunisian governorates, 7% of sheep without past abortions were seropositive for *C. burnetii* compared to 12% of small ruminants with previous abortions [49]. Another Tunisian study found that 19% of small ruminants with a history of abortion had *C. burnetii* detected by PCR analysis of birth products or vaginal secretions [45], and in South Africa, the pathogen was found by smear microscopy in aborted lamb fetuses from all of six sheep farms sampled [46].

Human cohorts comprising individuals with infective endocarditis in Sousse and Sfax, Tunisia, as well as Algiers, Algeria, have demonstrated *C. burnetii* as the causative pathogen in 1–3% of cases [51]–[53]. Two studies of febrile patients in Sousse, Tunisia, serologically identified acute Q fever in 2% and 9% of hospital admissions [54], [55]. Q fever was responsible for 5% of patients with acute febrile illness hospitalized in Bobo-Dioulasso, Burkina Faso [56] as well as 3% of pediatric and 8% of adult admissions for severe febrile illness at two referral hospitals in the Kilimanjaro Region of northern Tanzania [11]. In two studies of patients admitted for community-acquired pneumonia in Yaoundé and Douala, Cameroon, 6% and 9% of persons aged >15 years had serologically-confirmed acute Q fever [57], [58]. In these studies, Q fever was the third most common etiologic agent of pneumonia, after *Streptococcus pneumoniae* and *Mycoplasma pneumoniae*. At a major hospital in Cape Town, South Africa, *C. burnetii* was not found to be the cause of any pneumonia cases in a 92-patient cohort [59].

### Risk factors for *Coxiella burnetii* infection or disease

Among Nigerian cattle near Zaria, no difference in seropositivity was detected for cattle managed semi-intensively versus extensively [30], [31]. In Cameroon, positive associations were found between seropositivity and cattle aged >2 years, female animals, those seen grazing with buffalo, and those for which the owner's ethnic group was recorded as Mbororo or ‘other’ when compared to Fulbe [33], [60].

In the Egypt study linking human and animal populations, rural human residents were more likely to test seropositive than those in urban areas [27]. In the linked study from Chad, human Q fever serostatus did not correlate with the proportion of seropositive animals within respective nomadic camps, and camel breeders were at higher risk for Q fever seropositivity than cattle breeders [28]. In Ghana, children of illiterate mothers had a two-fold higher risk of seropositivity compared to those of literate mothers [41]. There was no association detected between *C. burnetii* seropositivity and HIV serostatus in pregnant Tanzanian women [44]. In the hospitalized patient cohort in northern Tanzania, there was no difference in prevalence of acute Q fever infection in HIV-infected compared to HIV-uninfected individuals [11], and all cases of community-acquired pneumonia in the surveys of hospitalized patients in Cameroon were in HIV uninfected individuals [57], [58]. No studies of risk factors for animal disease remained after quality assessment.

### *Coxiella burnetii* control efforts

No disease control studies were found by our search.

## Discussion

The serological data reviewed in this study reveal evidence of widespread *C. burnetii* infection in multiple species and multiple sites throughout Africa. However, despite evidence of the pervasiveness of this pathogen, we found only 17 studies that used appropriate case definitions to quantify disease due to C. *burnetii* in humans and animals. Risk factors for exposure in humans and animals have been identified in some settings, but apart from assessing for associations between HIV and Q fever, we found no other evaluation of the epidemiologic risk factors for acute disease in animals or humans. No descriptions of disease control programs appear in published literature. *C. burnetii* was first reported in Africa in 1947 [61], but since then, the quantity and quality of epidemiologic research for this pathogen has been limited. We identified no disease incidence estimates, and the majority of research undertaken has limited validity due to non-random sampling procedures. Further, only two investigations using random sampling procedures studied linked human and animal livestock populations.

The majority of animal prevalence studies surveyed cattle, sheep, and goats. Studies of herds in Northern Africa, the Sahel and South Africa's Transvaal Province suggest *C. burnetii* infection in multiple ruminant species. Seroprevalence of *C. burnetii* ≤13% is frequently demonstrated in cattle [27]–[29], [34], [35] and generally higher seroprevalence (11–33%) is often observed among small ruminants [27], [28], [36], [48]. However, this pattern was not observed in all studies [30]–[33]. Our review revealed wide variation in seroprevalence even in areas of close proximity such as Lake Chad, where seroprevalence in cattle ranged from 4% to 31% [28], [33]. This is consistent with findings of high regional variability within Europe [62] and highlights the importance of understanding risk factors which may operate at a local scale and may be subtle. For example, risk factor analysis revealed that cattle seropositivity differed by owner's ethnic group (Mbororo vs. Fulbe) in Cameroon, despite these groups' similar nomadic pastoralism [60]. Herds raised by unspecified ‘other ethnic groups’ had an even greater risk of infection, highlighting the need to further explore how different animal husbandry practices might modify infection risk for humans and animals. Further, the high seroprevalence observed in camels and the greater infection risk among Arab camel breeders compared to cattle breeders warrants further investigation of *C. burnetii* infection in camels and the potential risk factors related to camel husbandry practices [28].

Human seroprevalence was <8% [28], [42]–[44] with the exception of surveys in children and in the Nile Delta of Egypt [27], [39]–[41]. A recent survey in The Gambia found *C. burnetii* seroprevalence was highest in young children, although the reasons for this are still unclear [63]. In the only risk factor analysis among children found by our search, Ghanaian children with illiterate mothers were more likely to be seropositive than children with literate mothers, but no difference was attributable to other socioeconomic factors tested [41]. The study of Egyptians in close contact with animals reported a high overall seroprevalence (16%) with greater seropositivity among rural (22%) vs. urban (4%) residents [27]. In contrast, the only other linked human-animal survey studied nomads in Chad, and found a relatively low human seropositivity (1%), despite the high-risk behaviors of handling aborted animal materials and consuming raw cow's milk [28], which was shown to contain *C. burnetii* in 15–63% of samples from other African settings [30], [31], [38].

Only two studies [45], [46] used direct detection of *C. burnetii* in animal acute disease cases. The other studies investigating the potential role of *C. burnetii* as a cause of livestock abortions in Africa measured the seroprevalence in individuals with history of abortion as compared to individuals without history of abortion. Two cohort studies found no difference between *C. burnetii* seroprevalence in cattle with previous abortions compared to those without history of abortion [34], [50]. Similar studies in sheep and goats, by contrast, generally showed a higher *C. burnetii* seroprevalence in individuals with history of abortion compared to those without history of abortion [45], [47]–[49]. This serologic approach, however, has limited value for inferring causation in livestock abortion cases, which is compounded by non-random selection of control livestock without abortions in all but one study [30].

Q fever accounted for 2–9% of humans hospitalized for febrile illness in 3 different African sub-regions [11], [54]–[56]. Although Q fever was the third most common detected cause of community-acquired pneumonia at two of Cameroon's largest hospitals [57], [58], no cases of community-acquired pneumonia were attributed to *C. burnetii* after a year-long survey at a Cape Town hospital [59]. Interestingly, all of the Cameroonian patients were HIV-uninfected, and in the Tanzania severe febrile illness cohort there was no association between HIV serostatus and acute Q fever [11], [44]. The proportion of infective endocarditis cases in Africa attributed to *C. burnetii* was slightly lower than proportions found in European settings [64]. However, all endocarditis studies found by our review were conducted in Northern Africa [51]–[53], highlighting a key knowledge gap on the role of Q fever in endocarditis elsewhere on the continent.

We identified few studies that elucidated the epidemiologic risk factors for *C. burnetii* infection in humans and animals in Africa. This knowledge gap highlights the need for future studies that randomly sample linked human-animal populations in order to estimate seroprevalence and determine the dynamics of pathogen transmission. Such research requires large, representative samples as well as detailed surveys of herds and households from multiple locations, agricultural systems, and ethnic groups.

*C. burnetii* is clearly an important cause of human and animal disease in Africa, although illness and death have not been estimated at the population level. In animals, *C. burnetii* has been implicated as an etiologic agent of abortion in livestock from the most northern to the most southern reaches of the continent, but studies should include confirmatory microbiological and histological testing of abortion materials, descriptions of other disease sequelae, randomly sampled non-aborting controls, and tandem serological and bacterial shedding surveys to determine the rate of asymptomatic shedding. Estimates of economic losses due to decreases in milk production, fecundity, or birth weight are also needed. For humans, limited data suggest that Q fever frequently causes severe febrile illness in cohorts throughout Africa, yet no studies quantifying disease incidence, disability, or deaths at the population level exist. Further, aside from infective endocarditis studies, proportions and clinical features of conversion to chronic Q fever in African populations are absent.

### Limitations

Superseded geographic or biological terminology may have caused us to inadvertently miss pertinent research. The already remote chances of communicating with authors of older manuscripts were complicated by the absence of electronic contact information. We excluded arthropod vector studies, but surveys of invertebrates and non-domestic animals may contribute to knowledge about *C. burnetii* transmission. Comparisons between studies and sub-regions were restricted by changes in diagnostic methods over time, frequently small sample sizes, and the low total number of studies. The low number of studies for each research question and the heterogeneity of these studies precluded a more extensive quantitative analysis of the epidemiology of *C. burnetii* in Africa.

### Conclusions

To our knowledge, this is the first systematic review of the epidemiology of *C. burnetii* in Africa from a ‘One Health’ perspective. Taken together, these findings suggest: 1) exposure to *C. burnetii* is a common finding in many animal host species across Africa, but seroprevalence varies widely by species and location, and the risk factors underlying this variability are largely unknown; 2) *C. burnetii* has been implicated as a cause of livestock abortion and could be responsible for substantial economic burdens, but more rigorous studies are required to determine this and other sequelae of disease in animals; 3) risk factors for human exposure to Q fever are poorly understood, but a more detailed understanding of how human exposure in different communities is linked with animal infection patterns and animal husbandry practices is clearly needed; and 4) Q fever accounts for a notable proportion of undifferentiated human febrile illness and infective endocarditis but studies describing other acute or chronic disease manifestations are scarce. The picture is complex, but the existing literature suggests that *C. burnetii* is found across diverse settings in Africa and presents a real yet underappreciated threat to human and animal health throughout Africa.

**Key Points**

- Over the past 60 years, scores of studies have measured the prevalence of past or current infection of *C. burnetii* in humans and animals in Africa, but we found only 24 studies that used systematic, random sampling strategies, and of these only two studied linked human and animal populations.
- Our review identified only two studies of livestock abortion cases which sought to directly detect *C. burnetii* in animal birth products or in the female reproductive tract. Comprehensive studies of the etiology of livestock abortion in African contexts are required, and these studies should include *C. burnetii* in the battery of pathogens detection.
- Data from human cohort studies conducted in diverse settings in Africa show that Q fever accounts for 2–9% of severe fever cases, but more generalizable estimates of disease burden, such as incidence of Q fever, are lacking.
- Data on risk factors for *C. burnetii* transmission in Africa are limited, and further risk factor analyses oriented to the unique context of various African animal infection patterns and animal husbandry systems is warranted.

**Key Publications in the Field**

Blanc GM, L. A. and Maurica, A. (1947) Présence du virus de la ‘Q fever’ dans le Maroc méridional. Bull Acad Natl Med 131: 138–143.

Kaabia N, Rolain JM, Khalifa M, Ben Jazia E, Bahri F, et al. (2006) Serologic study of rickettsioses among acute febrile patients in central Tunisia. Ann N Y Acad Sci 1078: 176–179.

Nahed HG, Khaled AAM (2012) Seroprevalence of *Coxiella burnetii* antibodies among farm animals and human contacts in Egypt. J Am Sci 8: 619–621.

Schelling E, Diguimbaye C, Daoud S, Nicolet J, Boerlin P, et al. (2003) Brucellosis andQ-fever seroprevalences of nomadic pastoralists and their livestock in Chad. Prev Vet Med 61: 279–293.

Scolamacchia F, Handel IG, Fevre EM, Morgan KL, Tanya VN, et al. (2010) Serological patterns of brucellosis, leptospirosis and Q fever in *Bos indicus* cattle in Cameroon. PLoS One 5: e8623.

## Supporting Information

Table S1 **Studies of** ***Coxiella burnetii*** **seroprevalence in humans and animals in Africa with limited validity due to non-random sampling methods.** Note: The term ‘Domestic Animals’ was employed if >3 livestock or household animal species were investigated.
(DOC)
