# Supplementary material for: Epidemiology of Coxiella burnetii Infection in Africa: A OneHealth Systematic Review
Source: PLoS Negl Trop Dis. 2014 Apr 10;8(4):e2787. doi: 10.1371/journal.pntd.0002787 (PMC3983093; doi:10.1371/journal.pntd.0002787)
Supplement: Table S1 — Studies of Coxiella burnetii seroprevalence in humans and animals in Africa with limited validity due to non-random sampling methods. Note: The term ‘Domestic Animals’ was employed if >3 livestock or household animal species were investigated. (DOC) [file pntd.0002787.s001.doc]

**Table S1: Studies of *Coxiella burnetii* seroprevalence in humans and animals in Africa with limited validity due to non-random sampling methods**

| **Country** | **Species (Year of Publication)** | **Ref** |
| --- | --- | --- |
| **Northern Africa** |  |  |
| **Algeria** | Humans & Domestic Animals (1956, 1971)  Rodents (1963), Cattle (1970)  Humans, Sheep, Goats, Camels, Rodents & Arthropods (1984)  Humans (2009) |  |
| **Egypt** | Humans, Sheep, & Goats (1952, 2007)  Humans, Domestic & Wild Animals (1959)  Dogs & Ticks (1961)  Humans (1963, 1975, 1992, 1995)  Rodents & Ticks (1967)  Domestic Animals (1973)  Sheep & Goats (1978)  Dogs, Pigs & Rats (1989) |  |
| **Morocco** | Humans (1995) |  |
| **Sudan** | Humans, Domestic & Wild Animals (1959)  Humans (1963, 1995)  Cattle, Sheep & Goats (1962, 1988)  Camels (1972, 1987) |  |
| **Tunisia** | Humans, Goats, Sheep & Cattle (1954)  Rodents (1959)  Humans (1995) |  |
| **Western Africa** |  |  |
| **Burkina Faso** | Humans (1960, 1995, 2000) |  |
| **Cape Verde** | Humans & Domestic Animals (1987)  Rodents (1988)  Humans & Domestic Animals (1988)  Humans & Goats (1995)  Humans (1988, 1996) |  |
| **Ghana** | Humans (1968) |  |
| **Guinea Bissau** | Humans & Goats (1996) |  |
| **Ivory Coast** | Humans (1995)  Dogs (1998) |  |
| **Mali** | Humans (1995, 2005) |  |
| **Mauritania** | Humans (1998) |  |
| **Niger** | Cattle (2007)  Sheep & Goats (2008) |  |
| **Nigeria** | Cattle, Sheep & Goats (1977)  Camels (1980)  Domestic Birds (1982)  Horses & Pigs (1993) |  |
| **Senegal** | Humans (1964)  Rodents (1972)  Dogs (1998)  Cows’ Bulk Milk (2010)  Humans & Arthropods (2010) |  |
| **Togo** | Humans (1975) |  |
| **Middle Africa** |  |  |
| **Cameroon** | Humans (1953)  Humans & Cattle (1968) |  |
| **Central African**  **Republic** | Humans (1993, 1995)  Cattle (2004) |  |
| **Republic of Congo** | Humans, Cattle, Sheep & Goats (1954)  Cattle (1994)  Humans (1995) |  |
| **Eastern Africa** |  |  |
| **Comoros** | Humans (1995) |  |
| **Djibouti** | Cattle, Sheep & Goats (1994)  Humans (1995) |  |
| **Eritrea** | Humans (1964) |  |
| **Ethiopia** | Humans, Cattle, Sheep & Goats (1955)  Humans, Cattle, Sheep & Horses (1967)  Humans (1990) |  |
| **Kenya** | Rodents (1962)  Cattle (1973)  Humans & Cattle (1976) |  |
| **Madagascar** | Humans, Pigs, Sheep & Cattle (1959) |  |
| **Malawi** | Cattle (1989) |  |
| **Somalia** | Sheep & Goats (1978)  Humans (1995) |  |
| **Tanzania** | Humans, Cattle, Sheep, Goats & Game (1976)  Humans & Sheep (1996) |  |
| **Uganda** | Buffalo (2005) |  |
| **Southern Africa** |  |  |
| **South Africa** | Domestic Cats (1997) |  |
| **Zambia** | Cattle (1991)  Humans (1999) |  |
| **Zimbabwe** | Humans, Cattle, Goats & Dogs (1993)  Cattle (1993)  Ostriches (1996)  Domestic Cats (1997) |  |

**REFERENCES (TABLE S1)**

1. Neel R (1971) [Report on the work of the Pasteur Institute of Algeria during 1970]. / Rapport sur le fonctionnement de l'Institut Pasteur d'Algerie en 1970. Arch Inst Pasteur Alger 49: 141-185.

2. Dumas N (1984) [Rickettsiosis and chlamydiosis in Hoggar (Republic of Algeria): epidemiological sampling]. Bull Soc Pathol Exot Filiales 77: 278-283.

3. Lacheheb A, Raoult D (2009) Seroprevalence of Q-fever in Algeria. Clin Microbiol Infect 15 Suppl 2: 167-168.

4. Lacroix AC, Sayag A, Douard TH (1956) Serologic observations on Q fever in the Algerian region. Bull Soc Pathol Exot 49: 18-21.

5. Mailloux M (1963) [Microbiological survey on the rats of Algiers. III. Presence of *Rickettsia burneti*]. Bull Soc Pathol Exot Filiales 56: 149-156.

6. Schneider R, Le Corroller Y, de Lecubarri R (1970) [Human anti-rickettsial antibodies in domestic animals other than those which are classic reservoirs of viruses. Preliminary note: cattle]. Arch Inst Pasteur Alger 48: 203-205.

7. Taylor RM, Kingston JR, Rizk F (1959) Serological (complement-fixation) surveys for Q fever in Egypt and the Sudan, with special reference to to its epidemiology in areas of high endemicity. Arch Inst Pasteur Tunis 36: 529-556.

8. Sabban MS, Hussein N, Sadek B, Dahaby HE (1961) Q fever in dogs in Egypt. J Egypt Public Health Assoc 36: 147-152.

9. Hoogstraal H, Kaiser MN, Ormsbee RA, Osborn DJ, Hemly I, et al. (1967) *Hyalomma (Hyalommina) rhipicephaloides* Neumann (Ixodoidea: Ixodidae): its identity, hosts, and ecology, and *Rickettsia conori*, *R. prowazeki*, and *Coxiella burnetii* infections in rodent hosts in Egypt. J Med Entomol 4: 391-400.

10. Dirkvanpeenen PF, Reid TP (1963) A serological and stool survey of Bedouin tribesmen in the Western Desert of Egypt. Trop Geogr Med 15: 243-248.

11. McDade JE, Zaklama NS, Imam IZ, Wanees M (1973) Serological survey for Q fever in Egyptian domestic animals. J Egypt Public Health Assoc 48: 101-108.

12. Fiset P, Wisseman CLJ, El Batawi Y (1975) Immuologic evidence of human fetal infection with *Coxiella burnetii*. Am J Epidemiol 101: 65-69.

13. Schmatz HD, Krauss H, Viertel P, Ismail AS, Hussein AA (1978) [Seroepidemiological investigations in domestic ruminants from Egypt, Somalia and Jordan for the demonstration of complement fixing antibodies against Rickettsia and Chlamydia (author's transl)]. Acta Trop 35: 101-111.

14. Sixl W, Sebek Z, Kock M, Marth E, Withalm H (1989) [Serologic studies of domestic animals for listeriosis, Q-fever, and brucellosis in Cairo]. Geogr Med Suppl 3: 127-128.

15. Corwin A, Habib M, Olson J, Scott D, Ksiazek T, et al. (1992) The prevalence of arboviral, rickettsial, and Hantaan-like viral antibody among schoolchildren in the Nile River Delta of Egypt. Trans R Soc Trop Med Hyg 86: 677-679.

16. Botros BA, Soliman AK, Salib AW, Olson J, Hibbs RG, et al. (1995) *Coxiella burnetii* antibody prevalences among human populations in north-east Africa determined by enzyme immunoassay. J Trop Med Hyg 98: 173-178.

17. Mazyad SA, Hafez AO (2007) Q fever (*Coxiella burnetii*) among man and farm animals in North Sinai, Egypt. J Egypt Soc Parasitol 37: 135-142.

18. Meskini M, Beati L, Benslimane A, Raoult D (1995) Seroepidemiology of rickettsial infections in Morocco. Eur J Epidemiol 11: 655-660.

19. el Nasri M (1962) A serological survey for the detection of Q fever antibodies in the sera of animals in the Sudan. Bull Epizoot Dis Afr 10: 55-57.

20. Dirkvanpeenen PF, Gutekunst RR, Dietlein DR, Reid TP, Jr. (1963) Serological and skin test survey in a village of Central Sudan. Trans R Soc Trop Med Hyg 57: 297-305.

21. Harbi MS, el-Karim MH (1972) Serological investigation into Q fever in Sudanese camels (*Camelus dromedarius*). Bull Epizoot Dis Afr 20: 15-17.

22. Abbas B, el Zubeir AE, Yassin TT (1987) Survey for certain zoonotic diseases in camels in Sudan. Rev Elev Med Vet Pays Trop 40: 231-233.

23. Reinthaler FF, Mascher F, Sixl W, Arbesser CH (1988) Incidence of Q fever among cattle, sheep and goats in the Upper Nile province in southern Sudan. Vet Rec 122: 137.

24. Maurin J (1954) [Investigations on the existence of Q fever in Tunisia by the reaction of deviation of the complement]. Ann Inst Pasteur (Paris) 86: 68-75.

25. Juminer B (1959) Rattus norvegicus and Rickettsioses. Role of the rat as a probable virus reservoir for epidemic typhus and Q fever. Arch Inst Pasteur Tunis 36: 173-182.

26. Letaief AO, Yacoub S, Dupont HT, Le Cam C, Ghachem L, et al. (1995) Seroepidemiological survey of rickettsial infections among blood donors in central Tunisia. Trans R Soc Trop Med Hyg 89: 266-268.

27. Tissot-Dupont H, Brouqui P, Faugere B, Raoult D (1995) Prevalence of antibodies to *Coxiella burnetii*, *Rickettsia conorii*, and *Rickettsia typhi* in seven African countries. Clin Infect Dis 21: 1126-1133.

28. Sixl W, Sixl-Voigt B (1987) Research on a possible Q-fever infection in humans and animals on the Cape Verde Islands (Santa Cruz/Santiago, West Africa). J Hyg Epidemiol Microbiol Immunol 31: 472-474.

29. Brosch R, Miorini T, Buchrieser C, Buchrieser V, Sixl W (1988) Additional human Q-fever studies on the Cape Verde Islands--2nd report. Geogr Med Suppl 1: 103-106.

30. Brosch R, Sixl W, Buchrieser C, Buchrieser V, Miorini T (1988) Serological investigations in Q-fever and listeriosis of wild-living small mammals on the Cape Verde Islands. Geogr Med Suppl 1: 65-70.

31. Miorini T, Brosch R, Buchrieser C, Buchrieser V, Sixl W (1988) Further serological investigations in humans and domestic animals on the Cape Verde Islands (Q-fever, brucellosis, listeriosis, shigellosis, campylobacteriosis, yersiniosis, toxoplasmosis and chlamydia of PLT-group). Geogr Med Suppl 1: 19-31.

32. Kovacova E, Sixl W, Stunzner D, Urvolgyi J, Kazar J (1996) Serological examination of human and animal sera from six countries of three continents for the presence of rickettsial antibodies. Eur J Epidemiol 12: 85-89.

33. Russo V, D'Arrigo C (1968) Complement fixing antibodies against *Coxiella burnetii* in a sample of the population of Accra [Ghana]. Ann Med Nav (Roma) 73: 431-436.

34. Boni M, Davoust B, Tissot-Dupont H, Raoult D (1998) Survey of seroprevalence of Q fever in dogs in the southeast of France, French Guyana, Martinique, Senegal and the Ivory Coast. Vet Microbiol 64: 1-5.

35. Niang M, Parola P, Tissot-Dupont H, Baidi L, Brouqui P, et al. (1998) Prevalence of antibodies to *Rickettsia conorii Ricketsia africae*, *Rickettsia typhi* and *Coxiella burnetii* in Mauritania. Eur J Epidemiol 14: 817-818.

36. Zecchini M, Belli P, Harouna A, Vias G, Crimella C. Seroprevalence of brucellosis, chlamydiosis and Q fever in dairy cattle in the periurban area of Niamey. In: Camus E, Cardinale E, Dalibard C, Marinez D, Renard JF et al., editors. Proceedings of the 12th International Conference of the Association of Institutions for Tropical Veterinary Medicine (AITVM); 2007; Paris, France. Centre de Coopération Internationale en Recherche Agronomique pour le Développement (CIRAD).

37. Zecchini M, Harouna A, Cattaneo C, Belli P, Pecile A, et al. (2008) Seroprevalence of brucellosis, chlamydiosis, Q fever and mastitis in small ruminants in the urban and periurban area of Niamey (Niger). / Brucellosi, clamidiosi, febbre Q e mastiti negli allevamenti ovini e caprini in Niger. Large Anim Rev 14: 234-234.

38. Addo PB, Schnurenberger PR (1977) Q fever antibodies in food animals of Nigeria : a serological survey of cattle, sheep, and goats. Rev Elev Med Vet Pays Trop 30: 359-362.

39. Addo PB (1980) A serological survey for evidence of Q fever and in camels in Nigeria. Br Vet J 136: 519-521.

40. Addo PB (1982) A serological survey of a Q fever in indigenous domestic birds of Nigeria. Bull Anim Health Prod Afr 30: 211-213.

41. Ajuwape ATP, Falade S (1993) Serological evidence for Q fever in horses and pigs in Nigeria. Trop Vet 11: 79-81.

42. Moyen EN, Castets M, Boiron H (1964) [Preliminary survey on rickettsial diseases in Senegal]. Bull Soc Pathol Exot Filiales 57: 446-454.

43. Juminer B (1972) Study of anti-rickettsia agglutinins in *Cricetomys gambianus* in Dakar. Arch Inst Pasteur Tunis 49: 235-242.

44. Mediannikov O, Diatta G, Fenollar F, Sokhna C, Trape JF, et al. (2010) Tick-Borne Rickettsioses, Neglected Emerging Diseases in Rural Senegal. PLoS Negl Trop Dis 4.

45. Breurec S, Poueme R, Fall C, Tall A, Diawara A, et al. (2010) Microbiological quality of milk from small processing units in Senegal. Foodborne Pathog Dis 7: 601-604.

46. Schroter G, Loose B, Trojan H (1975) [Serological studies on the occurrence of rickettsial infections in Togo (author's transl)]. Tropenmed Parasitol 26: 323-328.

47. Giroud P, Capponi M, Roger F (1953) Serologic reactions to rickettsiae in meat handlers in Douala. Bull Soc Path Exot 46: 649-650.

48. Maurice Y, Fernagut R, Gerome R (1968) [Rickettsial diseases of North Cameroon; epidemiological study]. Rev Elev Med Vet Pays Trop 21: 341-349.

49. Belec L, Gresenguet G, Ekala MT, Jacob A, Vohito MD, et al. (1993) *Coxiella burnetii* infection among subjects infected with HIV type 1 in the Central African Republic. Eur J Clin Microbiol Infect Dis 12: 775-778.

50. Nakoune E, Debaere O, Koumanda-Kotogne F, Selekon B, Samory F, et al. (2004) Serological surveillance of brucellosis and Q fever in cattle in the Central African Republic. Acta Trop 92: 147-151.

51. Chantal J, Dorchies P, Legueno B (1994) A study on some zoonoses in Djbouti Republic .1. Ruminants from Djibouti slaughterhouse. Rev Med Vet (Toulouse) 145: 633-640.

52. Gray GC, Rodier GR, Matrasmaslin VC, Honein MA, Ismail EA, et al. (1995) Serologic evidence of respiratory and rickettsial infections among Somali refugees. Am J Trop Med Hyg 52: 349-353.

53. Taglieri G, Tresca G (1964) [Sero-epidemiological findings in a sample of the population of Asmara (Eritrea). VI. Investigation of complement-fixing antibodies against *Coxiella burnetii*]. Arch Ital Sci Med Trop Parassitol 45: 275-284.

54. Gutfreund R, Gelberg V, Chabaud MA (1955) Preliminary study of Q fever in Ethiopia. Bull Soc Pathol Exot 48: 451-453.

55. Reiss-Gutfreund RJ (1967) The epidemiology of Rickettsioses on the Ethiopian high-plateau. A six months survey, from October 1964 to April 1965. Amer J Trop Med Hyg 16: 186-190.

56. Abebe A (1990) Prevalence of Q fever infection in the Addis Ababa abattoir. Ethiop Med J 28: 119-122.

57. Heisch RB, Grainger WE, Harvey AEC, Lister G (1962) Feral aspects of rickettsial infections in Kenya. Trans R Soc Trop Med Hyg 56: 272-282.

58. Gossler R, Leyk W, Hunermund G (1973) [Serological studies on cattle in the Kabete area of Kenya. I. Occurrence of antibodies against parainfluenza-3, IBR and BVD viruses, Chlamydia and *Coxiella burnetii*]. / Serologische Untersuchungen bei Rindern im Einzugsgebiet von Kabete (Kenia) 1. Mitt.: Vorkommen von Antikorpen gegen Parainfluenza-3-, IBR-, BVD-Virus, Chlamydien und *Coxiella burnetii*. Berl Munch Tierarztl Wochenschr 86: 164-166.

59. Vanek E, Thimm B (1976) Q fever in Kenya. Serological investigations in man and domestic animals. East Afr Med J 53: 678-684.

60. Sureau P (1959) Serological investigation concerning Q fever in Madagascar. Arch Inst Pasteur Madagascar 27: 35-36.

61. Staley GP, Myburgh JG, Chaparro F (1989) Serological evidence of Q fever in cattle in Malawi. Onderstepoort J Vet Res 56: 205-206.

62. Hummel PH (1976) Incidence in Tanzania of CF antibody to *Coxiella burnetii* in sera from man, cattle, sheep, goats and game. Vet Rec 98: 501-505.

63. Kalema-Zikusoka G, Bengis RG, Michel AL, Woodford MH (2005) A preliminary investigation of tuberculosis and other diseases in African buffalo (*Syncerus caffer*) in Queen Elizabeth National Park, Uganda. Onderstepoort J Vet Res 72: 145-151.

64. Matthewman L, Kelly P, Hayter D, Downie S, Wray K, et al. (1997) Exposure of cats in southern Africa to *Coxiella burnetii*, the agent of Q fever. Eur J Epidemiol 13: 477-479.

65. Ghirotti M, Semproni G, De Meneghi D, Mungaba FN, Nannini D, et al. (1991) Sero-prevalences of selected cattle diseases in the Kafue flats of Zambia. Vet Res Commun 15: 25-36.

66. Okabayashi T, Hasebe F, Samui KL, Mweene AS, Pandey SG, et al. (1999) Short report: prevalence of antibodies against spotted fever, murine typhus, and Q fever rickettsiae in humans living in Zambia. Am J Trop Med Hyg 61: 70-72.

67. Rhode C, Kelly PJ, Raoult D (1993) Dairy cows as reservoirs of *Coxiella burnetii* in Zimbabwe. Cent Afr J Med 39: 208-210.

68. Kelly PJ, Matthewman LA, Mason PR, Raoult D (1993) Q fever in Zimbabwe. A review of the disease and the results of a serosurvey of humans, cattle, goats and dogs. S Afr Med J 83: 21-25.

69. Kelly PJ, Masanvi N, Cadman HF, Mahan SM, Beati L, et al. (1996) Serosurvey for *Cowdria ruminantium,* *Coxiella burnetii*, and Spotted fever group rickettsiae in ostriches (*Struthio camelus*) from Zimbabwe. Avian Dis 40: 448-452.
